# Design and Workspace Analysis of a Differential Motion Rotary Style Breast Interventional Robot

**DOI:** 10.1155/2020/8852228

**Published:** 2020-12-30

**Authors:** Yongde Zhang, Liyi Sun, Dexian Liang, Haiyan Du

**Affiliations:** ^1^Intelligent Machine Institute, Harbin University of Science and Technology, Harbin, China; ^2^Key Laboratory of Advanced Manufacturing and Intelligent Technology, Harbin University of Science and Technology, Harbin, China

## Abstract

**Introduction:**

Magnetic Resonance Imaging has better resolution for soft tissue; at the same time, the robot can work in a stable manner for a long time. MRI image-guided breast interventional robots have attracted much attention due to their minimally invasive nature and accuracy. In this paper, a hydraulic-driven MRI-compatible breast interventional robot is proposed to perform breast interventional procedure.

**Methods:**

First is the analysis of the design requirements of the hydraulic-driven MRI-compatible breast interventional robot, and then the design scheme is determined. Second, the three-dimensional model and the link frames are established. The workspace of the robot end point is solved by MATLAB/Simulink software. Then, the 3D printing technology is used to make a physical model of the MRI-compatible breast interventional robot. After assembly and debugging, the physical model is used for workspace verification, and the simulation result of the workspace shows that it is correct. Finally, the experimental research on the positioning error of the hydraulic drive is carried out, which established the theoretical foundation for the follow-up control research of the robot.

**Results:**

The positioning error has nothing to do with the motion distance, speed, and length of the selected tubing. The errors are 0.564 mm, 0.534 mm, and 0.533 mm at different distances of 40 mm, 80 mm, and 120 mm, respectively. The errors are 0.552 mm, 0.564 mm, and 0.559 mm at different speeds of 3 mm/s, 5 mm/s, and 8 mm/s, respectively. The errors are 0.564 mm, 0.568 mm, and 0.548 mm for different lengths of 0.5 m, 1 m, and 1.6 m, respectively. Then, the robot's working space on the *XOZ* plane and the *XOY* plane meets the conditions.

**Conclusion:**

The structure of a differential rotary breast interventional robot is determined, with the link frames assigned to the mechanism and the Denavit-Hartenberg parameters given. Workspace simulation of MRI-compatible breast interventional robot is done in MATLAB. The 3D printed MRI-compatible breast interventional robot is assembled and debugged to verify that its working space and positioning error meet the requirements.

## 1. Introduction

Recently, the number of women who have breast cancer disease increased significantly. It has reached first place in female malignancies [1–3]. The methods used for breast cancer patients during examinations have an impact on their detection rate and diagnosis rate [4]. However, Magnetic Resonance Imaging (MRI), compared to computed tomography (CT), can provide multiparameter and multisequence imaging with basically no limitations, high diagnostic efficiency, and important information for disease characterization and examination [5–7].

For breast cancer surgery which requires breast preservation, breast surgery treatments focus on individualized treatment and can have precise and microtraumatic treatment [8–10]. Therefore, accurate diagnosis of breast cancer must be performed first, which is a prerequisite for puncture biopsy.

Lately, robots can help people achieve satisfactory results in the medical field. This paper studies the breast interventional robot which is MRI-compatible, i.e., the nuclear magnetic imaging cannot interfere with the robot. The doctors can choose the optimal surgical path under the guidance of real-time images. At present, several MRI-compatible breast interventional robots have been introduced in the world.

Larson et al. designed a robot for tissue puncture. This device can only perform biopsy of one side of the breast. If bilateral breast disease detection needs to be done, it has to be repositioned manually [11–13]. Yang et al. proposed a four-degree-of-freedom robot with a compact parallel mechanism in 2011. The authors used signal-to-noise ratio experiments to verify the interference of the entire device on MRI imaging and found it interfered with imaging [14]. In 2008, Bricault et al. designed a light intervention robot, but the robot's movement accuracy on the patient's body surface can only be guaranteed within a certain distance [15–17]. Hu adopted a flexible drive to achieve compatibility between the drive device and the MRI environment [18]. In 2015, Lou used a cable drive to transmit the power of the motor to the robot joint in the MRI environment [19], but the cable drive is easily deformed, has low efficiency, and is difficult to control.

In summary, it can be inferred from the above statistical analysis that there are two key problems in breast interventional procedures: one is that it is impossible to achieve full biopsy of the breast tissue on both sides, and the other is that the driving method adopted by the robot interferes with imaging. In the nuclear magnetic environment, the hydraulic drive is a new flexible power transmission method, which transmits power to each joint of the robot through the tubing. In this paper, the design of the hydraulic-driven MRI-compatible breast interventional robot is developed from four aspects based on the modular idea. Secondly, the end-point workspace is solved, and a physical model is made to verify the correctness of the simulation results. The positioning error of hydraulic drive is studied to establish the foundation for the following research.

## 2. Structural Design of Breast Interventional Robot

### 2.1. Analysis of Functional Requirements of MRI-Compatible Breast Interventional Robot

The MRI instrument used in the hospital has an internal diameter of 600 mm and a length of 1.2~2 m. In order to ensure the high efficiency of the breast biopsy procedure, the patient is in the prone position. In this position, the effect of breathing motion on the breast can be ignored.

**Figure 1:**
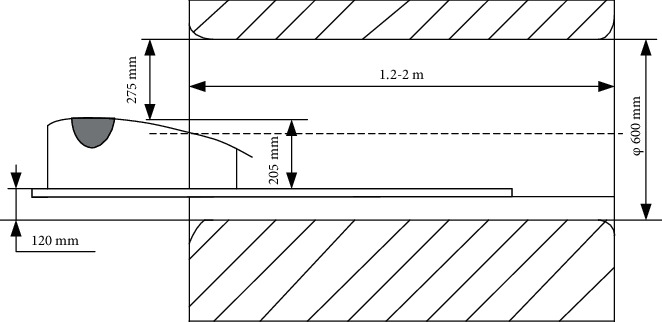
Available space for breast interventional robot under MRI.

Figure 1 shows the space diagram of the MRI and the prone support bracket. In order to prevent the collision between the breast interventional robot and the prone support bracket, it can be seen from the Figure 1 that the robot is in the bracket. The prone support bracket is 10 mm thick as the support surface. The robot cannot move more than 195 mm in the *Z* direction and 600 mm in the *Y* direction; the *X* direction is relatively unrestricted.

The breast is characterized by its softness and easy diffusion, which may lead to inaccurate puncture. When the robot performs interventional procedure, an auxiliary fixation mechanism is needed to restrict the fluidity of the breast tissue, then the lesion point of the breast tumour cannot be moved. This way, we ensure that the breast interventional robot can puncture the tumour lesion point. This is the first functional design requirement of the robot.

In order to achieve the shortest surgical path and avoid blood vessels when puncturing breast tumours, the breast interventional robots must have a positioning mechanism capable of comprehensive adjustment. This is the second functional design requirement of the robot.

The MRI-compatible breast interventional robot designed in this paper is used to perform puncture biopsy on the breast. The end-effector which can perform puncture biopsy needs to be installed. The biopsy mechanism designed in this paper is more convenient for the intelligent control of the whole process of puncture biopsy. This is the third functional design requirement of the robot.

According to the statistics of the survey report, the average size of a female breast is 145 × 100 mm. During the biopsy procedure, the patient lies prone on the prone support bracket, and the space at the end of the MRI-compatible breast interventional robot must reach or exceed 150 × 330 × 100 mm. This is the fourth functional design requirement of the robot.

### 2.2. System Overview

**Figure 2:**
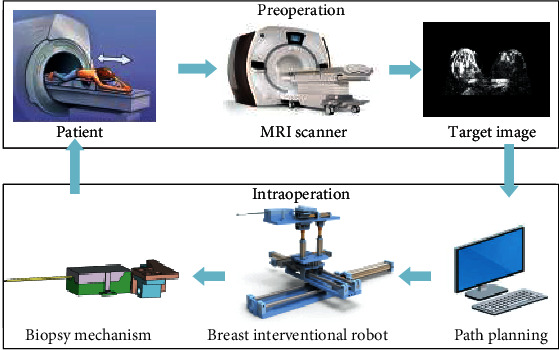
The overall structure of the breast intervention robot system.

According to the requirements of the MRI compatibility and minimally invasive surgery, a 6 DOF breast interventional robot was designed which includes a 3 DOF positioning module, a 2 DOF attitude adjustment module, and a puncturing module. At the same time, in order to restrict the flow of breast tissue during the puncture process and ensure the accuracy of the location of the puncture lesion, a rotary contraction-type breast tissue fixation device is designed. The overall structure of the breast intervention robot system is shown in Figure 2. The major components include a conventional MRI scanner system, workstation used for surgery planning (including the trajectory planning), preprocessing MRI image and the real-time monitoring of the operation process, and a breast intervention robot used to position the biopsy needle and to assist the surgeons for surgery. The entire procedure is under the MRI environment.

### 2.3. Rotary Contraction-Type Breast Tissue Fixing Device

**Figure 3:**
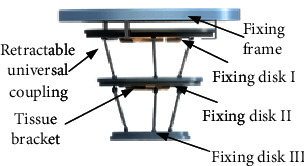
Structure of breast fixation device.

**Figure 4:**
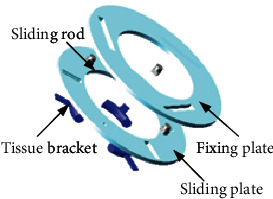
The fixed disk I.

In this paper, a rotary contraction-type breast tissue fixing device was designed for the fixation of breast tissue during breast interventional procedure. It mainly includes the fixing frame, fixing disk I, fixing disk II, fixing disk III, retractable universal coupling, sliding rod, and tissue bracket, as shown in Figure 3. Each fixing disk has a different diameter, from the largest diameter to the smallest diameter and is connected by a retractable universal coupling. There is a fixing plate, a sliding plate, and a sliding rod in each fixing disk. There are three sliding grooves on the fixing plate and the sliding plate, and the sliding rod moves in the sliding groove. By rotating the sliding plates in each fixing disk, the three tissue brackets can form different diameters, so as to better fix the breast tissue. It is easy to operate and flexible, as shown in Figure 4. The breast tissue fixation device itself has a tapered structure. In order to prevent the position of the retractable universal coupling from obstructing the biopsy of breast tissue, the entire device can be rotated axial along the central axis. Finally, the material of the tissue bracket was designed as rubber so that the patient can feel comfortable during the clamping process.

### 2.4. Design of Cartesian Coordinate Positioning Mechanism and Revolute Joint

**Figure 5:**
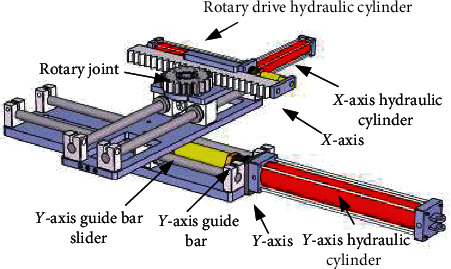
The breast interventional robot positioning mechanism and revolute joint connection.

Cartesian coordinate positioning mechanism is mainly composed of four parts, which are the support guide module, *X*-axis hydraulic cylinder, *Y*-axis hydraulic cylinder, and connecting mechanism. The revolute joint is made of a gear rack and rotary drive hydraulic cylinder. The combination of the Cartesian coordinate positioning mechanism and the revolute joint designed above makes the MRI-compatible breast interventional robot perform full-coverage puncture biopsy on both sides of the breast tissue, which is called a rotary-type structure. The positioning mechanism includes an *X*-axis hydraulic cylinder and a *Y*-axis hydraulic cylinder; the *Y*-axis hydraulic cylinder moving in the *Y* direction can meet the flexible switching of the robot between the bilateral breasts. In this way, the movement flexibility of the MRI-compatible breast interventional robot is greatly improved, and the control operation of the robot is simple. It is helpful for the successful operation of the breast interventional procedure, as shown in Figure 5.

### 2.5. Design of *Z*-Direction Positioning, Pitch Joint, and Biopsy Mechanism

In order to enable the MRI-compatible breast interventional robot to achieve the puncture biopsy of the tumour without being affected, it needs a mechanism that can change the position of the biopsy needle and realize the shortest and optimal puncture path.

**Figure 6:**
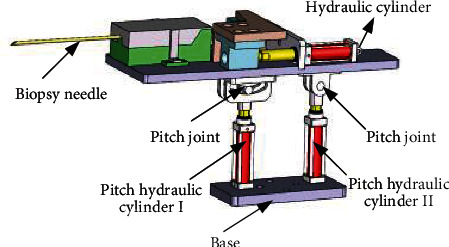
*Z*-direction positioning and pitching mechanism.

The biopsy mechanism is the end-effector of the breast interventional robot. The biopsy mechanism designed in this paper can perform the whole process of biopsy automatically, as shown in Figure 6. It mainly consists of a biopsy needle puncture mechanism, a biopsy needle mechanism connecting plate, and a moving slide. The drive is completed by a hydraulic cylinder.

Since the pitching joint is composed of two hydraulic cylinders in parallel, when the two hydraulic cylinders move at the same speed, the mechanism above the pitching joint can move up or down at the same time. When the two hydraulic cylinders move at different speeds, the mechanism above the pitching joint can form an up or down pitch angle, which can meet the needs of the pitching injection required for puncture. Combined with the rotary-type structure introduced above, the designed breast interventional robot was named as the differential motion rotary style breast interventional robot.

**Figure 7:**
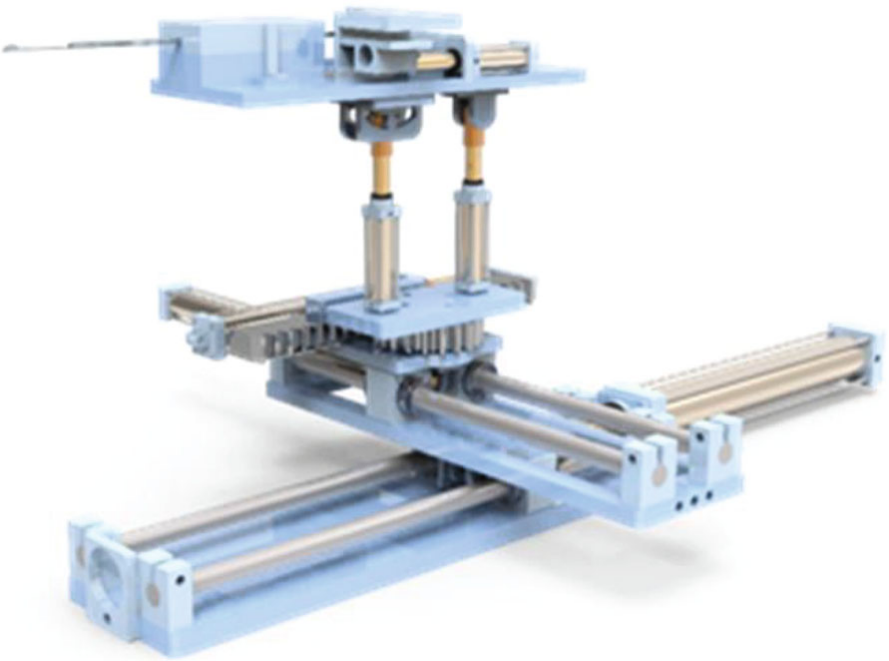
Hydraulic-driven MRI-compatible breast interventional robot structure.

The structure of the differential motion rotary style breast interventional robot is shown as Figure 7.

## 3. Motion Performance Analysis of Differential Rotary Breast Interventional Robot

### 3.1. Establishment of Link Frames of Breast Interventional Robot

**Figure 8:**
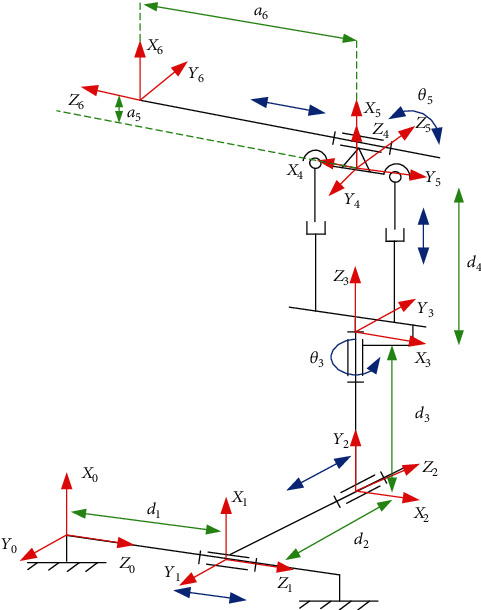
Link frames of breast intervening robot.

The Denavit-Hartenberg notation is adopted to establish the link frames of the robot and express the motion relationship of the mechanism [20]. For the robot with n joints, establish *n* + 1 frames first and take the base frame of the robot {0} as the origin. The frame of the robot on the I-th joint is denoted as frame {I} and the end-effector as frame {*n*} in the frames. According to the above description, the link frames are established for each link. The breast interventional robot has 6 degrees of freedom, which is divided into 4 prismatic joints and 2 revolute joints. Link frames of the differential rotary breast interventional robot are established, as shown in Figure 8.

According to the established link frames and link parameters of the differential rotary breast interventional robot, the parameters of each joint of the MRI-compatible breast interventional robot can be obtained, as shown in Table 1.

According to the established link frames and link parameters of the differential rotary breast interventional robot, the parameters of each joint of the MRI-compatible breast interventional robot can be obtained, as shown in Table 1.

#### 3.1.1. Forward Kinematics Analysis

To calculate the terminal joint I of the breast interventional robot, we only need to multiply the equation _*i*_^*i* − 1^*T* (*i* = 1, 2, ⋯, *n*) to get the transformation matrix of the base _*n*_^0^*T*

(1) Tn0=T10T21⋯Tnn−1.

Multiplying by_1_^0^*T*, _2_^1^*T*, _3_^2^*T*, _4_^3^*T*, _5_^4^*T*, and _6_^5^*T* can obtain the transformation matrix between the frame {6} and the base frame {0} in the end-effector of the breast interventional robot.

(2) T60=T10T21T32T4354T65T=nxoxaxpxnyoyaypynzozazpz0001.

Among them

(3) nx=sinθ5,ny=sinθ3cosθ5,nz=−cosθ3cosθ5,ox=0,oy=−cosθ3,oz=−sinθ3,ax=−cosθ5,ay=sinθ3sinθ5,az=−cosθ3sinθ5,px=a5sinθ5−d6cosθ5+d3+d4,py=a5sinθ3cosθ+5d6sinθ3sinθ−5d2,pz=−a5cosθ3cosθ5−d6cosθ3sinθ5+d1.

#### 3.1.2. Inverse Kinematics Analysis

We make the two ends of the forward kinematics formula (1) equal, that is,

(4) T60=T10nxoxaxpxnyoyaypynzozazpz0001=T10d1T21d2T32θ3T43d4T54θ5T65d6.

*θ*_3_ and *θ*_5_ are known parameters.

When to the matrix corresponding terms are equal, the following equation is obtained:

(5) $$\begin{array}{c} \left\{\begin{array}{c} \sin\theta_{5}=n_{x},\\ \sin\theta_{3}\cos\theta_{5}=n_{y},\\ -\cos\theta_{3}\cos\theta_{5}=n_{z},\\ 0=o_{x},\\ -\cos\theta_{3}=o_{y},\\ -\sin\theta_{3}=o_{z},\\ -\cos\theta_{5}=a_{x},\\ \sin\theta_{3}\sin\theta_{5}=a_{y},\\ -\cos\theta_{3}\sin\theta_{5}=a_{y},\\ a_{5}\sin\theta_{5}-d_{6}\cos\theta_{5}+d_{3}+d_{4}=p_{x},\\ a_{5}\sin\theta_{3}\cos\theta_{5}+d_{6}\sin\theta_{3}\sin\theta_{5}-d_{2}=p_{y},\\ -a_{5}\cos\theta_{3}\cos\theta_{5}-d_{6}\cos\theta_{3}\sin\theta_{5}+d_{1}=p_{z}. \end{array}\right. \end{array}$$

*θ*_3_ and *θ*_5_ can be directly solved by formula (5)

(6) $$\begin{array}{c} \theta_{3}=A\tan2\left(o_{z},o_{y}\right), \end{array}$$

(7) $$\begin{array}{c} \theta_{5}=A\tan2\left(n_{x},-a_{x}\right). \end{array}$$

In equation (7), we can get

(8) $$\begin{array}{c} d_{1}=p_{z}+a_{5}\cos\theta_{3}\cos\theta_{5}+d_{6}\cos\theta_{3}\sin\theta_{5},\\ d_{2}=-p_{y}+a_{5}\sin\theta_{3}\cos\theta_{5}+d_{5}\sin\theta_{3}\sin\theta_{5},\\ d_{4}=p_{x}-a_{5}\sin\theta_{5}+d_{5}\cos\theta_{5}-d_{3}. \end{array}$$

Thus, the value of *d*_1_, *d*_2_, *d*_4_, *θ*_3_, and *θ*_5_ is determined, and the joints can be driven to the specified position and orientation, so that the end execution can reach the specified position and orientation in the world frame.

The control of the breast interventional robot adopts a typical structure of a PID control system. As shown in formula (9), it can be seen that in a PID controller, the error signal *e* is used to generate the proportional, integral, and derivative actions and to generate the result signal. Weighting and summation to form the control signal *u*(*t*) are applied to the model. A mathematical description of the PID controller is

(9) $$\begin{array}{c} u\left(t\right)=k_{p}\left(e+\frac{1}{T_{i}}\int_{0}^{t}e\text{d}t\right)+T_{d}\frac{\text{d}e}{\text{d}t}. \end{array}$$

In the formula, *e* is the deviation between the desired position *θ*_*d*_ and the actual position *θ*, *k*_*p*_ is the proportional coefficient, *T*_*i*_ is the integral time constant, and *T*_*d*_ is the derivative time constant.

### 3.2. Workspace Analysis of Differential Rotary Breast Interventional Robot

**Figure 9:**
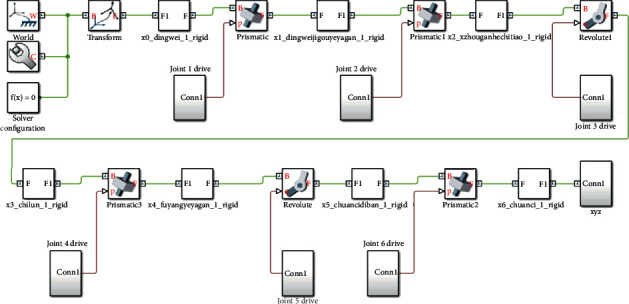
MRI-compatible breast interventional robot system simulation model.

The MRI-compatible breast intervention-robot model established in SOLIDWORKS software was imported into MATLAB/Simulink (MATLAB 2018, MathWorks) to obtain the simulation model of the robot, as shown in Figure 9. The breast interventional robot has 6 degrees of freedom and 8 joints, among which 2 prismatic joints and 2 revolute joints constitute a pitching platform in parallel, realizing the movement and pitching of the pitching platform with 2 degrees of freedom. According to the motion parameters of each joint of the designed breast interventional robot, a drive was added to the motion joint of the simulation model. Finally, the motion positions *X*, *Y*, and *Z* of the robot end-effector were the output.

**Figure 10:**
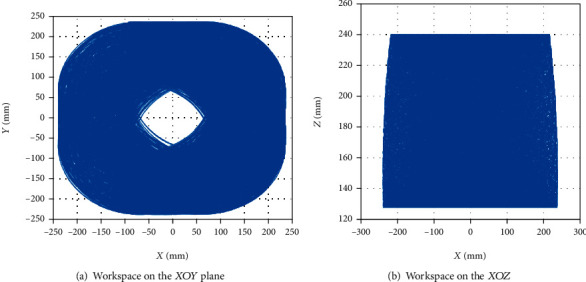
The projection of the MRI-compatible breast interventional robot workspace on the *XOY* plane and *XOZ* plane.

**Figure 11:**
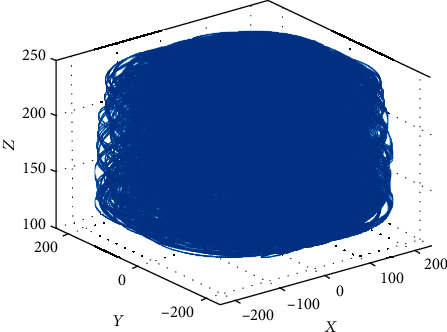
Simulation results of MRI-compatible breast interventional robot workspace.

The movement space of the robot is obtained according to the simulation result of the robot. As shown in Figure 10, it is a working space formed in the *XOY* and *XOZ* planes.  Figure 11 is a three-dimensional diagram of the space that the breast interventional robot can reach. Through analysis, it can be concluded that the working space of the breast interventional robot completely covers the necessary working space to meet the needs of the working space.

## 4. Positioning Error Measurement of Hydraulic-Driven Breast Interventional Robot

### 4.1. Composition of Hydraulic Drive Experiment System

The experimental platform of the hydraulic-driven breast interventional robot designed in this paper mainly includes an Arduino controller module, power module, sensor module, and external measurement module. According to the six DOF breast interventional robot designed above, all the joints are driven by the hydraulic cylinder to realize their movement, so all the joints of the robot can be controlled only by the control of the hydraulic cylinder.

**Figure 12:**
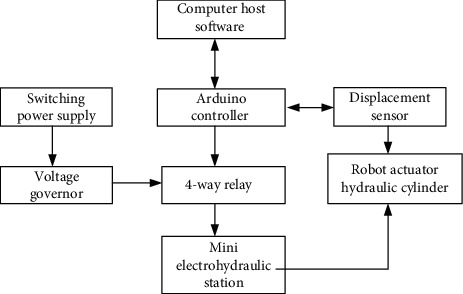
Hardware diagram of hydraulic robot control experimental system.

The hardware diagram of the control experimental system of the hydraulic driven robot is shown in Figure 12.

**Figure 13:**
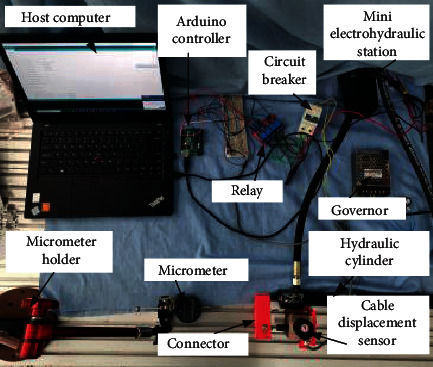
System wiring diagram.

The hydraulic drive system control experiment platform is shown in Figure 13.

The calibration distance of the hydraulic cylinder movement is set to 40 mm. Firstly, the movement of the hydraulic rod is controlled by the computer to stop at 40 mm and record the pulse number of the pull sensor. At the same time, the micrometer is placed at the stop position, so that the measuring head end point of the micrometer is in the same line position with the movement direction of the hydraulic cylinder and it can contact with the connecting block. The micrometer measures relative errors; when the hydraulic rod moves to 40 mm, this position is the marked zero position, leaving the micrometer in a compressed state and setting the reading to 0.00 mm.

**Figure 14:**
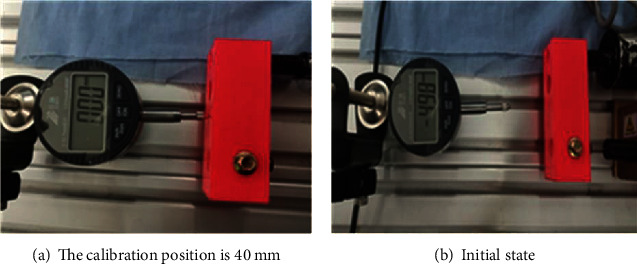
Calibration of hydraulic drive system.

The calibration of hydraulic drive system is shown in Figure 14. When the hydraulic cylinder moves to 40 mm, set the micrometer to zero for calibration. When the hydraulic cylinder returns to the origin, the micrometer returns to the normal state and the reading is -4.98 mm. After the calibration of the hydraulic rod is completed, the movement of the hydraulic cylinder is controlled by the host computer and the micrometer can record the movement error of each hydraulic cylinder.

Study of the positioning error of the hydraulic rod is divided into the following three cases. When the other conditions are the same, the effects of different moving distances, different speeds, and different tubing lengths on the positioning errors are studied.

### 4.2. Positioning Errors at Different Movement Distances

When the movement distance is 40 mm, the speed is 5 mm/s, and the length of the tubing is 50 cm, the positioning error is measured. The micrometer at the end of the probe is fixed at 40 mm and the micrometer should be -4.98 mm. The hydraulic rod is controlled to make it move a distance of 40 mm, so that it pushes the end of the micrometer probe. Then, the micrometer number is the positioning error of the hydraulic rod. The same method was measured for 30 times, and the data obtained are shown in Table 2.

Some parameters are needed to describe these errors. The average error is the mean value of the absolute value of the difference between the original data and the arithmetic mean. [21] The standard deviation reflects the degree of dispersion of a data set and reflects the degree of dispersion of the measured error. Root mean square error (RMSE) is the square root of the ratio of the square of the measured error to the number of observations [22]. It can evaluate the degree of change in the positioning error data. The smaller the value of the RMSE, the better the accuracy of the prediction model describing the experimental data. In this paper, the experimental verification is carried out from two aspects of precision and accuracy. Precision refers to the degree of consistency of the results obtained in multiple experiments, and accuracy refers to the error between the experimental results and the true value. Precision adopts the method of obtaining the standard deviation through multiple experiments, and the verification of accuracy is measured by the root mean square error between the experimental arrival position and the target position.

The mean error formula is

(10) $$\begin{array}{c} y=\frac{1}{n}\overset{n}{\underset{i=1}{\sum }}\left|x_{i}-\bar{x}\right|. \end{array}$$

The formula of the standard deviation is

(11) $$\begin{array}{c} y=\sqrt{\frac{{\left(x_{1}-x\right)}^{2}+{\left(x_{2}-x\right)}^{2}+\cdots +{\left(x_{n}-x_{1}\right)}^{2}}{n}}. \end{array}$$

The root mean square error formula is

(12) $$\begin{array}{c} \text{RSME}\left(X,h\right)=\sqrt{\frac{1}{m}\overset{m}{\underset{i=1}{\sum }}{\left(h\left(x^{\left(i\right)}\right)-y^{\left(i\right)}\right)}^{2}}. \end{array}$$

Calculate the average error, standard deviation, and root mean square error of the data in Table 2. The calculation results are 0.564, 0.664, and 0.743, which are within the accepted clinical performance tolerances of ±1 mm [23]. They indicate that the positioning error meets the use requirements.

We change the stroke of the hydraulic rod from the original 40 mm movement distance to 80 mm movement distance while other conditions remain the same, as shown in Table 3. We measure using the same method and collect 30 times of data. According to the data, the root mean square error is calculated, and RMSE is 0.639, the standard deviation is 0.642, and the average error is 0.534.

We change the stroke of the hydraulic rod to a movement distance of 200 mm while other conditions remain the same, as shown in Table 4. We measure in the same way and collected 30 times of data. The root mean square error is calculated as 0.603, the standard deviation is 0.604, and the average error is 0.553.

**Figure 15:**
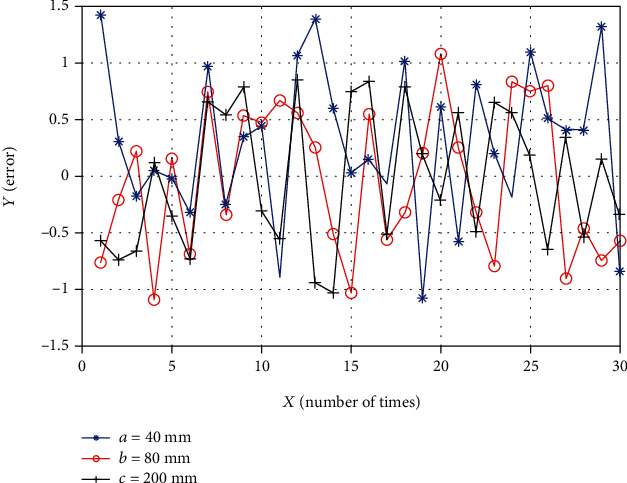
Line chart of error at three movement distances.

In summary, the three groups of motion distances are selected as the experimental objects. Their root mean square errors are between 1.0 and 1.2, and their standard deviations are between 0.55 and 0.64. It shows that the movement is stable. It can be obtained that the positioning error has nothing to do with the motion distance, so that the relationship between the three sets of errors can be displayed intuitively. A total of 90 sets of data from the three measurements are shown in Figure 15.

Table 5 shows the standard deviations at different movement distances, which are 0.664, 0.642, and 0.604, and the calculated average precision is 0.653 mm. The mean square error at different distances is 0.743, 0.639, and 0.603, and the calculated average accuracy is 0.658 mm.

### 4.3. The Influence of Different Motion Speeds on Positioning Error

We take the moving distance of the hydraulic cylinder as 40 mm and the length of the tubing as 50 cm, change the moving speed of the hydraulic rod, and measure its positioning error. The motion speed is changed to 3 mm/s and 8 mm/s. Combined with the positioning error of the velocity as 5 mm/s in Table 6, the influence of different motion speeds on the positioning error is analyzed.

The mean error, standard deviation, and root mean square error are 0.552, 0.630, and 0.634, respectively.

When measuring at an 8 mm/s speed, as shown in Table 7, the error is obtained and the mean error, standard deviation, and root mean square error are calculated, which are 0.559, 0.600, and 0.617, respectively. Comparing with the root mean square error of Table 2, it can be seen that the root mean square error is between 1.0 and 1.2 and the standard deviation is between 0.55 and 0.64. It means that the positioning error has nothing to do with speed.

**Figure 16:**
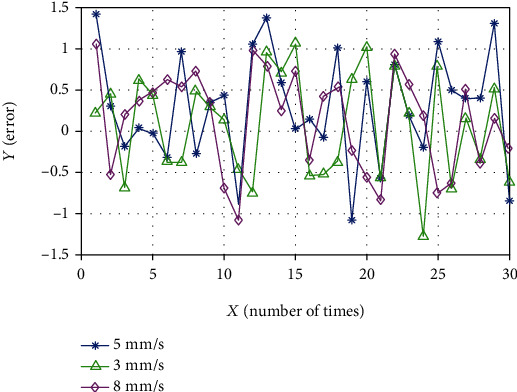
Line chart of error at different movement speeds.

The two sets of measured data are combined with the data in Table 2 to plot the positioning errors under three speeds, as shown in Figure 16.

Table 8 shows the standard deviations at different speeds, which are 0.630, 0.664, 0and .600, and the calculated average precision is 0.639 mm. The mean square error at different distances is 0634, 0.734, and 0.617, and the calculated average accuracy is 0.660 mm.

### 4.4. The Effect of Different Tubing Lengths on Positioning Accuracy

The effects of different tubing lengths on positioning errors were studied. The tubing lengths of 0.5 m, 1 m, and 1.6 m were selected to measure positioning errors. We select the speed of 5 mm/s and the motion stroke of 40 mm, then measure the positioning error of the tubing lengths of 1 m and 1.6 m. The influence of the tubing length on the positioning error was studied by combining the data in Table 2. The mean error, standard deviation, and root mean square error were 0.568, 0.559, and 0.612, respectively, as shown in Table 9.

Table 10 shows the positioning error of the hydraulic rod at 1.6 m tubing. The mean error of the 1.6 m tubing is 0.548, the standard deviation is 0.563, and the root mean square error is 0.582.

**Figure 17:**
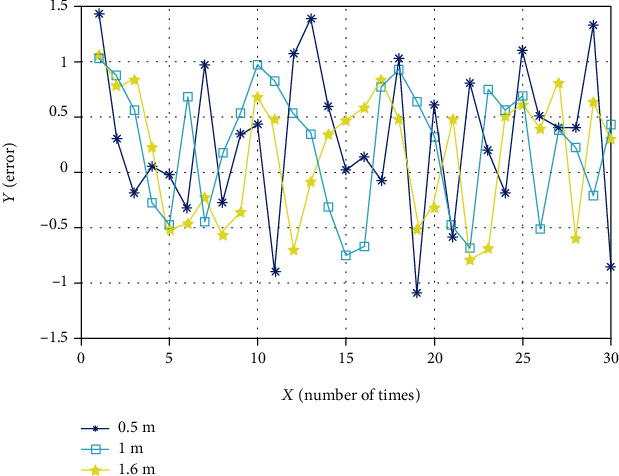
Line chart of error at different tubing.

According to Table 2, it can be seen that the root mean square error is between 1.0 and 1.2, and the standard deviation is between 0.55 and 0.64.  Figure 17 shows the line chart of error at different tubing lengths. It can be seen that the positioning error has nothing to do with the length of the selected tubing.

Table 11 shows the standard deviations at different tubing lengths, which are 0.664, 0.659, and 0.563, and the calculated average precision is 0.600 mm. The mean square error at different distances is 0.743, 0.612, and 0.582, and the calculated average accuracy is 0.643 mm.

## 5. Robot Workspace Validation Analysis

### 5.1. *XOZ* Plane Workspace Verification

**Figure 18:**
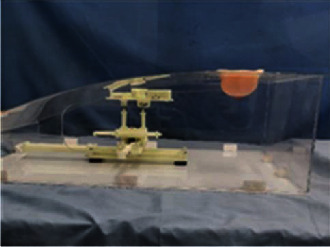
Physical model of breast interventional robot.

According to the analysis, the specific size of the space that the end point of the breast interventional robot must reach or exceed is 150 × 330 × 100 mm. The specific work area is obtained by simulating the work space of the breast intervention machine. According to the research of Zhang on several materials that can be used in the nuclear magnetic environment, the designed breast interventional robot is made of polytetrafluoroethylene and 3D printing technology [24]. Nonmagnetic stainless steel materials are selected for the biopsy needle and spring. The physical model of the breast interventional robot is shown in Figure 18.

**Figure 19:**
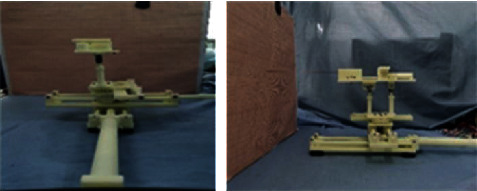
Set the zero point coordinates of the robot.

We verify the working space of the robot by manipulating the axes of the robot and recording the extreme positions it can reach. Firstly, we verify the *XOZ* plane and make it reach the limit position of the bottom left, top left, bottom right, and top right to form a rectangle. We record its position coordinates on the coordinate board and measure the distance between each vertex and verify its reachable space. We set the zero coordinate of the robot, as shown in Figure 19.

**Figure 20:**
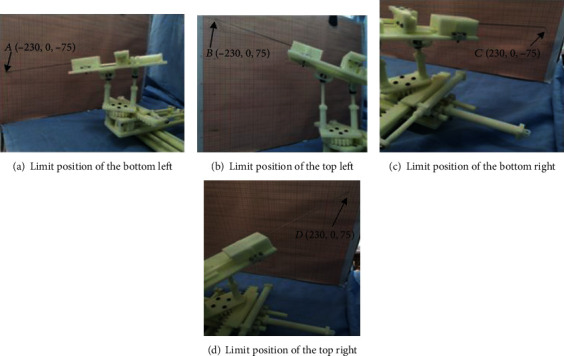
Vertex position.

We operate the robot to reach each vertex and record its position on the coordinate board. Then, we record the position coordinate *A* (−230, 0, −75), *B* (−230, 0, 75), *C* (230, 0, −75), and *D* (230, 0, 75) of each vertex, as shown in Figure 20.

**Figure 21:**
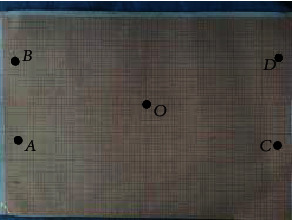
The position of each vertex in the coordinate board.

**Figure 22:**
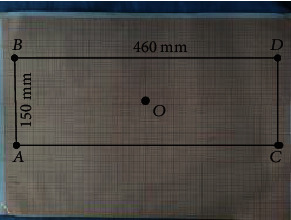
The workspace formed by connecting vertices.

Figure 21 shows the position of each point on the coordinate paper. From Figure 22, we can see that *AB* = 150 mm and *AC* = 460 mm. It can be seen that the working space meets the conditions.

### 5.2. *XOY* Plane Workspace Verification

**Figure 23:**
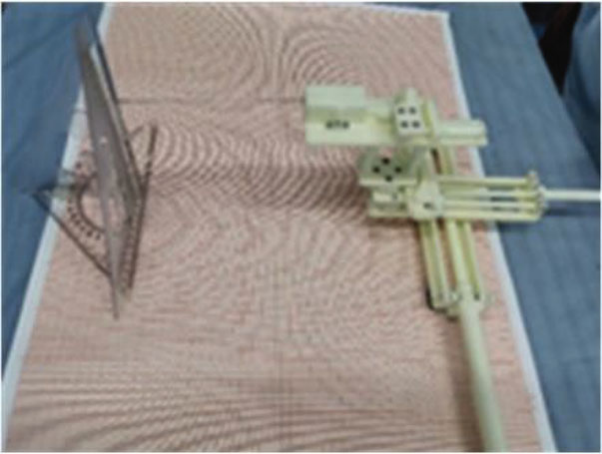
*XOY* plane set zero coordinate.

The same method is adopted to measure its vertex position and take six points in total. The coordinates of its vertex are recorded. First, the coordinates of its zero point are set, as shown in Figure 23.

**Figure 24:**
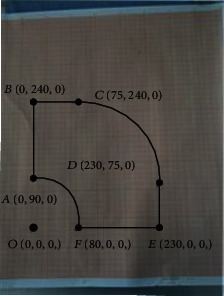
The position of each vertex in the coordinate board.

We operate the robot to each vertex and record its position on the coordinate board and then record the position coordinates of each vertex, *A* (0, 90, 0), *B*(0, 240, 0), *C*(75, 240, 0), *D*(230, 75, 0), *E*(230, 0, 0), *F*(80, 0, 0), as shown in Figure 24.

**Figure 25:**
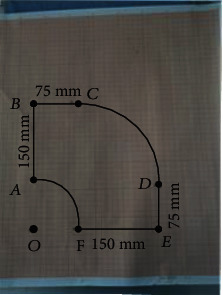
The workspace formed by connecting vertices.

Figure 25 shows a quarter of the working space of the robot on the *XOY* plane, so it can be seen that the whole working space of the robot on the *XOY* plane meets the conditions.

## 6. Discussion

With the development of surgical treatment of breast cancer, the treatment has been developing towards being minimally invasive, functional, and aesthetic, achieving the needs of individualization, safety, and precision. Therefore, it can be applied to all kinds of individuals. At the same time, it must be able to diagnose breast cancer, improve the detection rate and diagnosis rate, and reduce the cost of breast biopsy. At present, the specific steps of traditional breast biopsy interventional surgery are as follows: (1) firstly, MRI scanning is performed to establish the three-dimensional model of the lesion area; (2) after the imaging is completed, the patient is pushed out of the MRI and the doctor delivers the MRI markers through the biopsy needle; (3) afterwards, it is necessary to check whether the markers have reached the ideal position, and then the patient needs to be pushed into the MRI scanner again; (4) when the markers reach the ideal position, we perform puncture sampling and sample storage; and (5) after the biopsy operation, the MRI markers stored in the body can be used for subsequent chemotherapy tests. Through the above steps, the same process is repeated many times and the position of the lesion and the marker point cannot be seen in real time. The patient suffers a long time of torture and pain, and the progress of the biopsy operation is slow. Long operation times lead to high labor intensity of the doctors, and the accuracy of the acupuncture is not high. The therapeutic effect mainly depends on the personal experience and level of doctors.

The preliminary clinical experience of the medical robot is positive, and it has higher accuracy compared with the traditional handheld method [25, 26]. The designed breast interventional robot can not only reduce the pain of patients but also reduce the workload of doctors. At the same time, the efficiency and quality of the surgery are improved, which have a beneficial impact on the continued development of accurate breast cancer diagnosis and treatment.

In this paper, we have designed a MRI-compatible breast interventional robot. The breast interventional robot is placed in the MRI, and the doctor can choose the optimal surgical path under the guidance of real-time images. The rotary contraction-type breast tissue fixing device designed according to the shape of the human breast is used to fix the breast tissue during the biopsy process. The robot realizes positioning and navigation for puncture biopsy, so as to perform breast biopsy surgery comfortably and accurately. It is verified through experiments that the average precision of the robot driving joints under different movement distances is 0.66385 mm, and the average accuracy is 0.7433 mm. The average precision at different movement speeds is 0.66385 mm, and the average accuracy is 0.7433 mm. The average precision under different tubing lengths is 0.66385 mm, and the average accuracy is 0.7433 mm. Compared with the clinical manual needle insertion, this result can meet its accuracy requirements. According to the current research of the breast interventional robot system, breast examination can be done by ultrasound images or MRI images. Due to the limitations of poor imaging quality and low resolution of ultrasound images, the positioning accuracy of the breast interventional robot is affected. For example, the accuracy of the ultrasound-guided breast interventional robot designed by Abayazid et al. is 3 mm [27], and that of Kaya et al. is 1.17 mm [28]. At the same time, many scholars began to study MRI-compatible breast interventional robot and the driving method also needs to be compatible with the MRI environment. The current MRI-compatible driven methods have their own shortcomings. They use flexible cables or wire drives, which are easy to deform, have low efficiency, and are difficult to control. When driven by an ultrasonic motor, etc., it will adversely affect the image. When air pressure is used as a power device, its speed reliability and safety are poor. It not only affects the positioning accuracy and has high noise but also has an impact on the quality of MRI-guided breast interventional surgery. This article proposes a new and flexible power transmission method that is hydraulic driven in the nuclear magnetic environment. The power is transmitted to the joints of the robot through the tubing. The driving device is placed outside the nuclear magnetic instrument, which ensures that the nuclear magnetic instrument does not contain a motor and other metal materials.

The goal of future work is to integrate the hydraulic drive system and the robot system for further experiments. The experimental platform only carries out the drive control of the robot single joint. Therefore, in order to meet the needs of robot application, the multidegree of freedom robot motion which combines advanced robot technology and measurement technology will be studied.

## 7. Conclusions

In this paper, the structure of a differential rotary breast interventional robot was determined by analyzing the MRI-compatible breast interventional robot. The correlation analysis was carried out to ensure the rationality of the robot mechanism design. Then, one of the prismatic joints of the robot was selected and the experimental platform was built to measure its positioning error and working space, and the analysis was carried out.

## Figures and Tables

**Table 1:** Denavit-Hartenberg parameters of the robotic arm.

Rod *i*	*a*_*i*−1_	*α*_*i*−1_	*d*_*i*_	*θ*_*i*_
1	0	0°	*d*_1_	0°
2	0	90°	*d*_2_	90°
3	0	90°	*d*_3_	*θ*_3_
4	0	0°	*d*_4_	180°
5	0	90°	0	*θ*_5_
6	*a*_5_	90°	*d*_6_	0°

*a*_5_ is the known quantity *a*_5_ = 10 mm.

**Table 2:** Positioning error of hydraulic rod with 40 mm movement distance.

Times	Error (mm)	Times	Error (mm)	Times	Error (mm)
1	1.43	11	-0.89	21	-0.58
2	0.31	12	1.07	22	0.81
3	-0.18	13	1.39	23	0.20
4	0.05	14	0.60	24	-0.19
5	-0.02	15	0.03	25	1.10
6	-0.32	16	0.15	26	0.51
7	0.97	17	-0.07	27	0.41
8	-0.27	18	1.02	28	0.41
9	0.35	19	-1.08	29	1.32
10	0.44	20	0.61	30	-0.84

**Table 3:** Positioning error of hydraulic rod with 80 mm movement distance.

Times	Error (mm)	Times	Error (mm)	Times	Error (mm)
1	-0.76	11	0.67	21	0.25
2	-0.21	12	0.56	22	-0.32
3	0.22	13	0.26	23	-0.79
4	-1.09	14	-0.51	24	0.84
5	0.16	15	-1.03	25	0.75
6	-0.69	16	0.55	26	0.80
7	0.75	17	-0.56	27	-0.90
8	-0.34	18	-0.32	28	-0.46
9	0.54	19	0.20	29	-0.75
10	0.47	20	1.08	30	-0.57

**Table 4:** Positioning error of hydraulic rod with 200 mm movement distance.

Times	Error (mm)	Times	Error (mm)	Times	Error (mm)
1	-0.57	11	-0.55	21	0.56
2	-0.74	12	0.85	22	-0.49
3	-0.66	13	-0.94	23	0.65
4	0.12	14	-1.03	24	0.56
5	-0.35	15	0.75	25	0.19
6	-0.73	16	0.84	26	-0.65
7	0.66	17	-0.51	27	0.34
8	0.54	18	0.79	28	-0.54
9	0.79	19	0.20	29	0.16
10	-0.30	20	-0.21	30	-0.34

**Table 5:** Precision and accuracy of different movement distances.

	40 mm	80 mm	200 mm	Average value
Average error	0.564	0.534	0.553	0.550
Standard deviation	0.664	0.642	0.604	0.653
RMSE	0.743	0.639	0.603	0.658

**Table 6:** Positioning error of hydraulic rod at 3 mm/s speed.

Times	Error (mm)	Times	Error (mm)	Times	Error (mm)
1	0.23	11	-0.46	21	-0.56
2	0.45	12	-0.75	22	0.82
3	-0.69	13	0.96	23	0.22
4	0.63	14	0.71	24	-1.29
5	0.44	15	1.08	25	0.79
6	-0.36	16	-0.54	26	-0.69
7	-0.38	17	-0.53	27	0.16
8	0.49	18	-0.37	28	-0.34
9	0.31	19	0.64	29	0.51
10	0.15	20	1.02	30	-0.61

**Table 7:** Positioning error of hydraulic rod at 8 mm/s speed.

Times	Error (mm)	Times	Error (mm)	Times	Error (mm)
1	1.06	11	-1.09	21	-0.83
2	-0.53	12	0.99	22	0.94
3	0.20	13	0.79	23	0.56
4	0.37	14	0.26	24	0.20
5	0.47	15	0.74	25	-0.76
6	0.63	16	-0.34	26	-0.64
7	0.54	17	0.42	27	0.51
8	0.74	18	0.55	28	-0.39
9	0.36	19	-0.23	29	0.16
10	-0.69	20	-0.56	30	-0.21

**Table 8:** Precision and accuracy at different speeds.

	3 mm/s	5 mm/s	8 mm/s	Average value
Average error	0.552	0.564	0.559	0.558
Standard deviation	0.630	0.664	0.600	0.639
RMSE	0.634	0.743	0.617	0.660

**Table 9:** Positioning error of hydraulic rod at 1 m tubing.

Times	Error (mm)	Times	Error (mm)	Times	Error (mm)
1	1.02	11	0.82	21	-0.47
2	0.86	12	0.54	22	-0.68
3	0.56	13	0.35	23	0.75
4	-0.27	14	-0.31	24	0.55
5	-0.48	15	-0.76	25	0.69
6	0.68	16	-0.67	26	-0.51
7	-0.45	17	0.76	27	0.39
8	0.19	18	0.93	28	0.22
9	0.54	19	0.64	29	-0.21
10	0.97	20	0.32	30	0.44

**Table 10:** Positioning error of hydraulic rod at 1.6 m tubing.

Times	Error (mm)	Times	Error (mm)	Times	Error (mm)
1	1.06	11	0.48	21	0.18
2	0.78	12	-0.71	22	-0.79
3	0.84	13	-0.08	23	-0.68
4	0.23	14	0.35	24	0.51
5	-0.52	15	0.47	25	0.62
6	-0.46	16	0.58	26	0.41
7	-0.22	17	0.84	27	0.82
8	-0.58	18	0.48	28	-0.61
9	-0.36	19	-0.52	29	0.64
10	0.69	20	-0.32	30	0.31

**Table 11:** Precision and accuracy at different tubing.

	0.5 m	1 m	1.6 mm	Average value
Average error	0.564	0.568	0.548	0.560
Standard deviation	0.664	0.559	0.563	0.600
RMSE	0.743	0.612	0.582	0.643

## Data Availability

The data used to support the findings of this study are available from the corresponding author upon request.
